# A comprehensive in silico and invitro analysis revealed the diagnostic, prognostic and therapeutic potential of GNAI family genes in colon adenocarcinoma (COAD)

**DOI:** 10.1186/s41065-025-00523-3

**Published:** 2025-08-16

**Authors:** Bei Wang, Fan Zhu, Yingying Chen

**Affiliations:** 1 Anorectal surgery, The People’s Hospital of Danyang, Danyang Jiangsu, 212300 China; 2 Department of General Surgery, Ezhou Central Hospital, Hubei, 436000 China; 3 Department of Gastroenterology, Affiliated Hospital of Xuzhou Medical University, Xuzhou Jiangsu, 221000 China

**Keywords:** COAD, GNAI genes, Prognosis, Therapeutic target

## Abstract

**Introduction:**

Guanine nucleotide-binding protein alpha inhibiting activity polypeptides (GNAI1, GNAI2, and GNAI3) play critical roles in cell cycle regulation, intracellular signaling, and immune modulation. However, their contribution to colorectal adenocarcinoma (COAD) pathogenesis remains poorly defined. This study aimed to comprehensively evaluate the diagnostic, prognostic, and therapeutic relevance of GNAI genes in COAD through integrated in silico and in vitro analyses.

**Methods:**

mRNA and protein expression profiles of GNAI1, GNAI2, and GNAI3 were analyzed using TCGA, OncoDB, HPA databases, and RT-qPCR analysis across COAD cell lines. Genetic and epigenetic alterations were assessed using UALCAN, cBioPortal, and GSCA databases. Prognostic significance was evaluated through Kaplan–Meier survival and GENT2 databases. Potential miRNA regulators were identified via TargetScan and quantified using TaqMan assays. Immune interactions, immune infiltration, and drug sensitivity were examined using TISIDB and GSCA platforms. Functional effects of GNAI1 and GNAI2 overexpression were tested in SW480 and HCT116 cell lines using proliferation, colony formation, and wound healing assays.

**Results:**

GNAI1, GNAI2, and GNAI3 were significantly downregulated in both COAD tissues and cell lines. This downregulation correlated with promoter hypermethylation, CNV deletions, and reduced patient survival. ROC analysis indicated better diagnostic potential, particularly for GNAI2 (AUC = 0.83). Pathway analysis revealed suppression of DNA damage and cell cycle regulatory pathways and activation of EMT-related signaling. Upregulated miRNAs—hsa-miR-133a-3p-1, hsa-miR-138-5p, and hsa-miR-141-3p—were identified as direct regulators, exhibiting strong diagnostic capacity. Immune profiling showed that GNAI genes were differentially expressed across immune subtypes, negatively correlated with immune inhibitors, and positively associated with stimulators. Overexpression of GNAI1 and GNAI2 significantly inhibited COAD cell proliferation, clonogenic potential, and migration.

**Conclusion:**

This study reveals the tumor-suppressive function of GNAI1, GNAI2, and GNAI3 in COAD through genetic, epigenetic, and miRNA-mediated regulation. Their downregulation is associated with poor prognosis, altered immune landscape, and therapy resistance. Restoration of GNAI function represents a promising avenue for diagnostic and therapeutic intervention in colorectal cancer.

**Clinical trial number:**

None.

**Supplementary Information:**

The online version contains supplementary material available at 10.1186/s41065-025-00523-3.

## Introduction

Colorectal cancer (CRC) is a major global health burden, ranking as the third most commonly diagnosed cancer and the second leading cause of cancer-related mortality worldwide [1–3]. Among its histological subtypes, colon adenocarcinoma (COAD) accounts for the vast majority of cases [2, 4]. According to the Global Cancer Observatory (GLOBOCAN) 2024 data, over 1.9 million new cases of CRC and more than 935,000 associated deaths were recorded globally, reflecting the urgent need for improved strategies for early diagnosis, prognosis, and therapy [1, 5]. Although significant progress has been made in surgical techniques, chemotherapeutic regimens, and targeted therapies, the prognosis for patients with advanced or metastatic COAD remains dismal, with five-year survival rates dropping sharply beyond the localized stage [6, 7]. The clinical management of COAD continues to face challenges due to late-stage diagnosis, recurrence, metastasis, and resistance to conventional treatment modalities [8, 9].

Advances in molecular oncology have highlighted the importance of understanding the genetic and epigenetic alterations driving COAD progression [10–12]. In this context, heterotrimeric G proteins and their regulatory subunits have emerged as critical modulators of oncogenic signaling [13]. The guanine nucleotide-binding protein alpha inhibiting (GNAI) subfamily—comprising GNAI1, GNAI2, and GNAI3—encodes the α-subunits of inhibitory G proteins involved in G protein-coupled receptor (GPCR) signaling [14]. These proteins primarily act by inhibiting adenylyl cyclase activity, thereby reducing cyclic AMP (cAMP) levels and altering downstream signaling cascades involved in cell proliferation, migration, and survival [14].

Recent studies have increasingly implicated the GNAI subfamily in the regulation of tumor initiation, progression, and metastasis across a wide range of malignancies. Notably, GNAI2 has been reported to function as a tumor suppressor in glioblastoma, where it inhibits cell proliferation and survival by negatively regulating the phosphatidylinositol 3-kinase (PI3K)/AKT signaling axis—a pathway known to be hyperactivated in glioma and associated with poor patient prognosis [15, 16]. In contrast, in pancreatic ductal adenocarcinoma, GNAI2 exerts pro-tumorigenic effects by activating the extracellular signal-regulated kinase (ERK1/2) signaling cascade, thereby enhancing tumor cell proliferation, epithelial-to-mesenchymal transition (EMT), and invasive potential [17]. This dual role highlights the tissue-specific and context-dependent functions of GNAI2 in cancer biology [18, 19]. GNAI1, another member of the subfamily, has also been shown to possess tumor-suppressive functions [18]. In hepatocellular carcinoma, overexpression of GNAI1 significantly reduced metastatic capability by interfering with the RhoA-ROCK signaling pathway, a known regulator of cytoskeletal remodeling and cell motility [20]. Additionally, GNAI1 has been reported to modulate autophagy and apoptosis-related signaling pathways, including JNK and AMPK, in various experimental models, suggesting broader roles in cellular homeostasis under oncogenic stress [20]. Furthermore, dysregulated expression or mutational alteration of GNAI genes has been associated with adverse clinical outcomes in breast, gastric, lung, and ovarian cancers [21–23]. For instance, low GNAI2 expression correlates with trastuzumab resistance in HER2-positive breast cancer [24], while GNAI1 overexpression has been linked to poor survival and chemoresistance in advanced-stage ovarian tumors [25]. GNAI3, though less studied, has gained attention for its roles in tumor development and immune regulation. In triple-negative breast cancer (TNBC), GNAI3 promotes tumor proliferation and metastasis by facilitating Wnt/β-catenin signaling [26]. In hepatocellular carcinoma, high GNAI3 expression has been associated with poor prognosis, potentially through modulation of TGF-β signaling and interaction with immune checkpoint pathways [27]. GNAI3 also regulates lysosomal activity and vesicle trafficking, processes that are increasingly recognized as important in tumor survival and drug resistance [28]. Furthermore, GNAI3 has been shown to influence the expression of PD-L1 in certain cancer models, suggesting a possible role in immune escape mechanisms [27].

In the present study, we comprehensively investigated the role of GNAI family members in COAD through a combination of in silico [29, 30] and in vitro [31] approaches. Publicly available transcriptomic datasets and clinical metadata were analyzed to assess the expression patterns, diagnostic utility, and prognostic significance of GNAI genes in COAD patients [32, 33]. Furthermore, to validate and explore their functional roles, we overexpressed GNAI1 and GNAI2 in COAD cell lines and performed a series of cellular assays including, proliferation, colony formation, and wound healing assays [34, 35]. These findings aim to advance our understanding of GNAI-mediated signaling in COAD biology and provide a foundation for the development of novel molecular biomarkers or therapeutic targets.

## Methodology

### Cell lines

Nine COAD cell lines (HCT116, SW480, SW620, HT-29, LoVo, Caco-2, DLD-1, RKO, and LS174T) and six normal human colon epithelial cell lines (CCD 841 CoN, FHC, CCD-18Co, NCM460, CRL-1790, and CCD-112CoN) were purchased. Cells were cultured in either Dulbecco’s Modified Eagle Medium (DMEM; Cat. No. 11965092) or RPMI-1640 medium (Cat. No. 11875119), supplemented with 10% heat-inactivated fetal bovine serum (FBS; Cat. No. 10082147) and 1% Penicillin-Streptomycin (Cat. No. 15140122), as recommended for each specific cell line. Cells were maintained in a humidified incubator at 37 °C with 5% CO₂, and the medium was replaced every 2–3 days. Upon reaching 80–90% confluence, cells were detached using TrypLE Express (Cat. No. 12604013) or 0.25% Trypsin-EDTA (Cat. No. 25200056), and passaged for expansion or harvested for downstream analyses. All cell lines were authenticated using short tandem repeat (STR) profiling and routinely tested for mycoplasma contamination using the MycoAlert™ PLUS Mycoplasma Detection Kit (Lonza).

### Quantitative real-time PCR (RT-qPCR) analysis

Total RNA was extracted from COAD and normal colon cell lines using the PureLink™ RNA Mini Kit (Thermo Fisher Scientific, Cat. No. 12183018 A) following the manufacturer’s protocol. RNA concentration and purity were assessed using a NanoDrop™ 2000 spectrophotometer (Thermo Fisher Scientific). Complementary DNA (cDNA) was synthesized from 1 µg of total RNA using the High-Capacity cDNA Reverse Transcription Kit (Thermo Fisher Scientific, Cat. No. 4368814). RT-qPCR was performed using PowerUp™ SYBR™ Green Master Mix (Thermo Fisher Scientific, Cat. No. A25742) on a QuantStudio™ 5 Real-Time PCR System (Thermo Fisher Scientific). Gene-specific primers were used for amplification purposes (Table 1), and each reaction was carried out in triplicate under standard cycling conditions. Relative gene expression was quantified using the 2^^−ΔΔCt^ method, with GAPDH serving as the endogenous control for normalization across all samples. This normalization step corrects for sample-to-sample variation in RNA input, reverse transcription efficiency, and other technical variables, ensuring accurate and reliable quantification of target gene expression. The stability of GAPDH expression was confirmed across experimental groups prior to its use as a reference gene.

**Table 1:** Primer sequences used for RT-qPCR analysis

Gene	Forward Primer (5’→3’)	Reverse Primer (5’→3’)
GAPDH	ACCCACTCCTCCACCTTTGAC	CTGTTGCTGTAGCCAAATTCG
GNAI1	AGCACTGAGTGACTACGACCTG	GGATGTATCTGTAAACCACTTGTTG
GNAI2	CATCTTCTGCGTAGCCTTGAGC	GATGGACGTGTCTGTGAACCAC
GNAI3	CACTTCACCTGTGCCACAGACA	GTCTGGTCTCAACACTCCACAC
Vimentin	CAGAAAGTTTTCCACCAAAG	ACTGAACCTGACCGTACAAAATGTGAGCAATTCTGCTT
E-cadherin	AGGAAATGGCTCGTCACCTTCGTGAATA	GGAGTGTCGGTTGTTAAGAACTAGAGCT

### mRNA expression validation analysis

The mRNA expression levels of GNAI1, GNAI2, and GNAI3 in COAD tissue samples were analyzed using transcriptomic data from The Cancer Genome Atlas (TCGA) COAD cohort. Expression data were retrieved and processed via the Gene Set Cancer Analysis (GSCA) (https://bioinfo.life.hust.edu.cn/GSCA/) [36] and OncoDB (https://www.oncodb.org/) [37] platforms. Stage-specific expression profiles were evaluated to examine differential expression patterns across COAD clinical stages. Additionally, Gene Set Enrichment Analysis (GSEA) was conducted using the GSCA database to investigate the functional relevance and enrichment of GNAI genes in COAD.

### Protein expression validation analysis

The protein expression levels of GNAI1, GNAI2, and GNAI3 in COAD and normal colon tissues were analyzed using immunohistochemistry (IHC) images retrieved from the Human Protein Atlas (HPA) database (https://www.proteinatlas.org/) [38]. Representative IHC staining images were examined to compare protein expression patterns between COAD and normal tissue samples. The intensity and localization of staining were used as indicators of protein expression levels.

### Genetic and epigenetic profiling

Genetic and epigenetic alterations of GNAI1, GNAI2, and GNAI3 in COAD were analyzed using multiple publicly available databases. Promoter methylation data were obtained from the UALCAN database (https://ualcan.path.uab.edu/) [38] to assess DNA methylation levels in tumor versus normal tissues. Somatic mutation frequencies and types were evaluated using the cBioPortal database (https://www.cbioportal.org/) [39]. Copy number variation (CNV) data were retrieved from the GSCA database.

### Survival and pathway activation analysis

Survival analysis of GNAI1, GNAI2, and GNAI3 in COAD was performed using the KM Plotter database (https://kmplot.com/analysis/) [27], which correlates gene expression levels with overall patient survival using Kaplan–Meier plots and log-rank tests. Meta-analysis of hazard ratios across multiple COAD cohorts was conducted through the GENT2 database (http://gent2.appex.kr) [40]. To assess the involvement of GNAI genes in oncogenic signaling, pathway activity analysis was also carried out using GSCA database.

### miRNA regulatory and diagnostic analysis

Putative miRNAs targeting the 3′ UTR regions of GNAI1, GNAI2, and GNAI3 were predicted using the TargetScan database (https://www.targetscan.org/vert_80/) [41]. The expression levels of the identified miRNAs—hsa-miR-133a-3p-1, hsa-miR-138-5p, and hsa-miR-141-3p—were evaluated using RT-qPCR in nine COAD and six normal colon cell lines. miRNA expression was quantified using TaqMan™ Advanced miRNA Assays and normalized to U6 snRNA.

### Immune landscape and drug sensitivity analysis

The immune regulatory role of GNAI1, GNAI2, and GNAI3 in COAD was assessed using the TISIDB database (http://cis.hku.hk/TISIDB/) [42]. Gene expression levels were analyzed across COAD immune subtypes (C1–C6), and their correlations with immune inhibitory and stimulatory molecules were evaluated. Additionally, immune infiltration and drug sensitivity analysis were performed using the GSCA database.

### Protein–protein interaction and functional enrichment analysis

PPI networks for GNAI1, GNAI2, and GNAI3 were constructed using the Pathway Commons database (https://www.pathwaycommons.org/) [43], which integrates interaction data from multiple pathway resources. Functional enrichment analysis was carried out using the Database for Annotation, Visualization and Integrated Discovery (DAVID) (https://david.ncifcrf.gov/) [44], where cellular component, molecular function, and biological process categories were analyzed based on Gene Ontology (GO) terms. Additionally, pathway enrichment analysis was conducted using the Kyoto Encyclopedia of Genes and Genomes (KEGG) to identify relevant biological pathways associated with GNAI genes and their interacting partners.

### Induction of GNAI1 and GNAI2 overexpression in SW480 and HCT116 cell lines

To evaluate the functional impact of GNAI1 and GNAI2 overexpression in COAD, we transfected SW480 and HCT116 cell lines with GNAI1 and GNAI2 expression plasmids (Thermo Fisher Scientific) using Lipofectamine™ 3000 Transfection Reagent (Cat. No. L3000015) according to the manufacturer’s protocol. Forty-eight hours post-transfection, total protein was extracted using RIPA buffer supplemented with protease and phosphatase inhibitors (Thermo Fisher Scientific, Cat. No. 78440). Protein concentrations were measured using the Pierce™ BCA Protein Assay Kit (Cat. No. 23225). Equal amounts of protein were resolved by SDS-PAGE, transferred onto PVDF membranes, and probed with primary antibodies against GNAI1 Cat. No. PA1-1000), GNAI2 (Cat. No. PA1-28931), and GAPDH (loading control) (Cat. No. TAB1001), followed by detection with HRP-conjugated secondary antibodies and Pierce™ ECL Western Blotting Substrate (Cat. No. 32106).

### Cell proliferation, colony formation, and wound healing assays

Cell proliferation was assessed using the Cell Counting Kit-8 (CCK-8) assay (Dojindo Molecular Technologies, distributed by Thermo Fisher Scientific, Cat. No. A311-01) following the manufacturer’s instructions. Briefly, cells were seeded in 96-well plates at a density of 3,000–5,000 cells per well in 100 µL of complete medium and allowed to adhere overnight. At the indicated time points (e.g., 24, 48, and 72 h), 10 µL of CCK-8 solution was added to each well and incubated for 2 h at 37 °C in a humidified incubator with 5% CO₂. The absorbance was measured at 450 nm using a microplate reader to determine cell viability.

Colony formation assays were conducted by seeding 500 cells per well in 6-well plates containing 2 mL of complete growth medium. Cells were incubated at 37 °C in a humidified incubator with 5% CO₂ for 10–14 days, with medium refreshed every 3–4 days. Once visible colonies (≥ 40 cells per colony) had formed, cells were gently washed with PBS, fixed with 4% paraformaldehyde for 15 min at room temperature, and then stained with 0.5% crystal violet solution for 20 min. Excess stain was washed off with distilled water, and plates were air-dried. Colonies were imaged and manually counted under a light microscope, and the number of colonies per well was quantified for comparison between groups.

For wound healing assays, a scratch was introduced in a confluent monolayer using a sterile 200 µL pipette tip. Cells were washed with PBS and incubated in serum-free medium. Wound closure was imaged at 0 and 24 h using a phase-contrast microscope, and migration was quantified using ImageJ software. All experiments were performed in triplicate.

### Statistical analysis

All experiments were conducted in triplicate unless otherwise specified. Quantitative data were expressed as mean ± standard deviation (SD). Differences between two groups were analyzed using the unpaired two-tailed Student’s t-test, while comparisons among multiple groups were assessed using one-way ANOVA followed by Tukey’s post hoc test. Tukey’s correction was applied to account for multiple comparisons in the ANOVA framework, ensuring control of the family-wise error rate. Receiver Operating Characteristic (ROC) curve analysis was performed to evaluate diagnostic performance, and AUC values were reported. Kaplan–Meier survival curves were compared using the log-rank test. P*-value < 0.05, P**-value < 0.01, and P***-value < 0.001 were considered statistically significant. Statistical analyses and graphical representations were performed using GraphPad Prism (v9.0) and R software (v4.2.1).

## Results

### Downregulation of GNAI genes in COAD cell lines

**Fig. 1:**
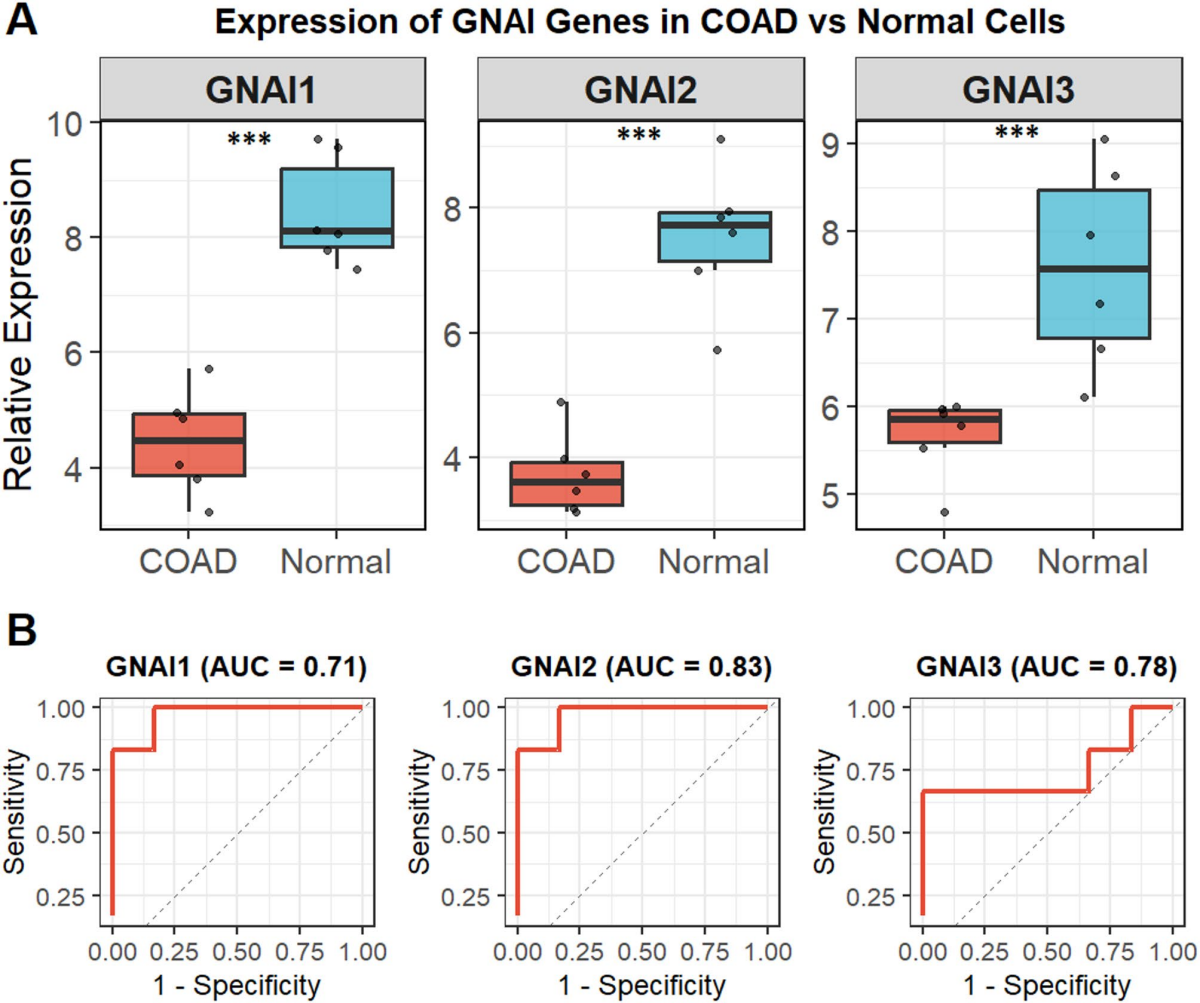
Downregulation and diagnostic potential of GNAI1, GNAI2, and GNAI3 in COAD cell lines. (**A**) RT-qPCR analysis showing the relative mRNA expression levels of GNAI1, GNAI2, and GNAI3 in nine COAD cell lines compared to six normal colon cell lines. (**B**) Receiver Operating Characteristic (ROC) curve analysis assessing the diagnostic performance of GNAI1, GNAI2, and GNAI3 in distinguishing COAD from normal cell lines. P***-value < 0.001

The expression levels of GNAI1, GNAI2, and GNAI3 were analyzed in nine COAD and six normal cell lines using RT-qPCR. The results showed that all three genes exhibited significantly (*p*-value < 0.001) lower expression in COAD cells compared to normal cells (Fig. 1A). To assess the diagnostic potential of these genes, ROC curve analysis was performed, and the Area Under the Curve (AUC) values were calculated. GNAI1 achieved an AUC of 0.71, indicating modest diagnostic accuracy (Fig. 1B). GNAI2 showed the highest AUC of 0.83, reflecting a relatively better, though not exceptional, diagnostic performance, while GNAI3 had an AUC of 0.78, suggesting moderate diagnostic utility slightly lower than that of GNAI2 (Fig. 1B). These findings suggest that while GNAI genes, particularly GNAI2, may offer some diagnostic relevance, their standalone clinical utility requires further validation and may benefit from integration with additional biomarkers.

### mRNA expression validation analysis of GNAI genes across COAD tissue samples

**Fig. 2:**
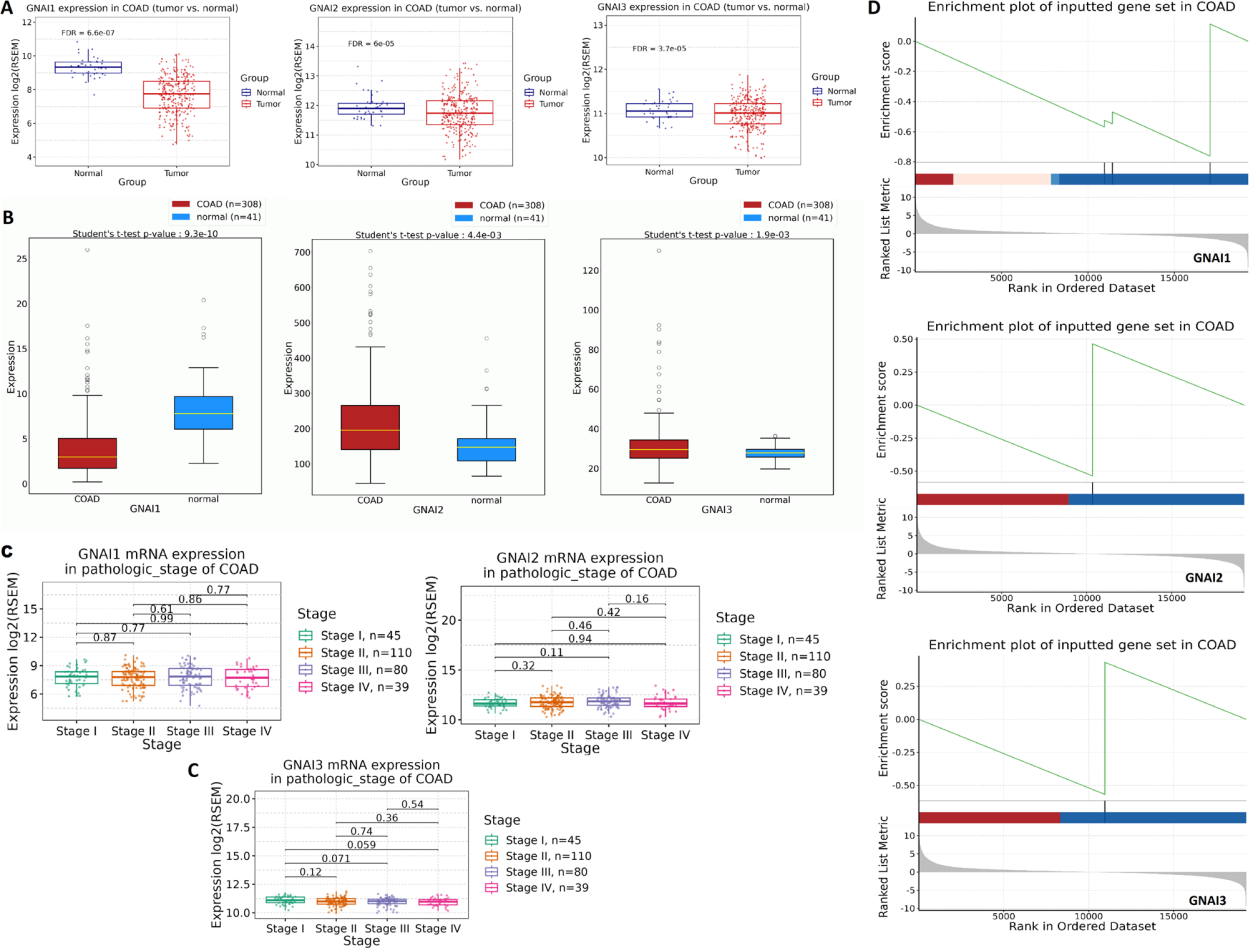
Validation of GNAI gene downregulation and stage-wise expression analysis in COAD tissues. (**A**–**B**) mRNA expression levels of GNAI1, GNAI2, and GNAI3 in COAD vs. normal tissue samples from the TCGA COAD cohort analyzed using the GSCA and OncoDB databases. All three genes were significantly downregulated in COAD tissues. (**C**) Stage-wise expression analysis of GNAI genes across COAD samples. (**D**) Gene Set Enrichment Analysis (GSEA) from the GSCA database showing significant enrichment of GNAI1, GNAI2, and GNAI3 gene sets in COAD tissues. *P*-value < 0.05

The expression of GNAI genes (GNAI1, GNAI2, and GNAI3) in COAD tissue samples was validated using data from the TCGA COAD cohort through the GSCA and OncoDB databases. The results revealed significant downregulation of GNAI1, GNAI2, and GNAI3 in COAD tissue samples compared to normal tissue samples (Fig. 2A-B). Further analysis of GNAI gene expression across different stages of COAD showed a gradual decline in GNAI1 expression from Stage I to Stage IV (*p* = 0.87), although this trend did not reach statistical significance. For GNAI2 (*p* = 0.16) and GNAI3 (*p* = 0.54), expression levels did not significantly differ between stages, indicating that their expression patterns are not strongly associated with disease stage progression (Fig. 2C). These findings suggest that while all three genes are downregulated in COAD overall, stage-specific variation is limited and not statistically significant. Finally, Gene Set Enrichment Analysis (GSEA) using the GSCA database validated the significant enrichment of GNAI1, GNAI2, and GNAI3 in COAD compared, highlighting their stronger association with COAD pathology (Fig. 2D).

### Protein expression validation analysis of GNAI genes across COAD tissue samples

**Fig. 3:**
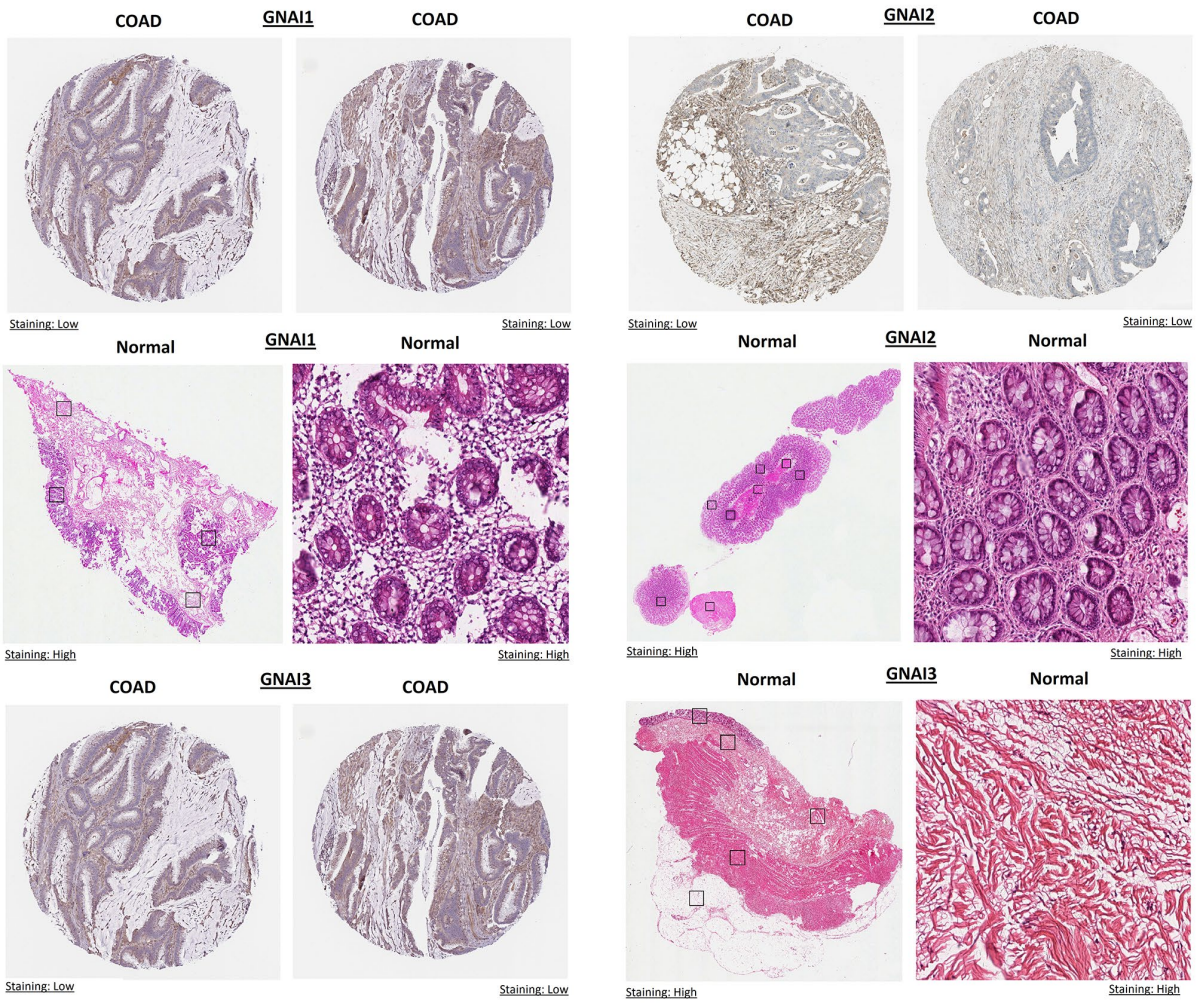
Protein expression validation of GNAI1, GNAI2, and GNAI3 in COAD tissues using the HPA database. Immunohistochemistry (IHC) images from the HPA database reveal differential protein expression of GNAI1, GNAI2, and GNAI3 in COAD and normal tissues. All three genes exhibit weak staining in COAD tissues, indicating low protein expression, whereas strong staining is observed in normal tissues, confirming higher protein expression

The protein expression of GNAI genes (GNAI1, GNAI2, and GNAI3) was validated in COAD tissue samples using data from the HPA database. The results show that GNAI1, GNAI2, and GNAI3 exhibit low protein expression in COAD tissues, as indicated by weak staining patterns in the respective images for each gene (COAD images) (Fig. 3). In contrast, normal tissues show high protein expression, as demonstrated by the stronger staining patterns in the normal samples (Fig. 3). The expression of GNAI1 in normal tissue is notably higher compared to COAD, with clear, high-intensity staining observed. Similarly, GNAI2 and GNAI3 also show significantly higher protein expression in normal tissues compared to COAD tissues, as evidenced by the strong staining in the normal samples versus the weak staining in COAD samples (Fig. 3).

### Genetic and epigenetic profiling of GNAI family genes in COAD

**Fig. 4:**
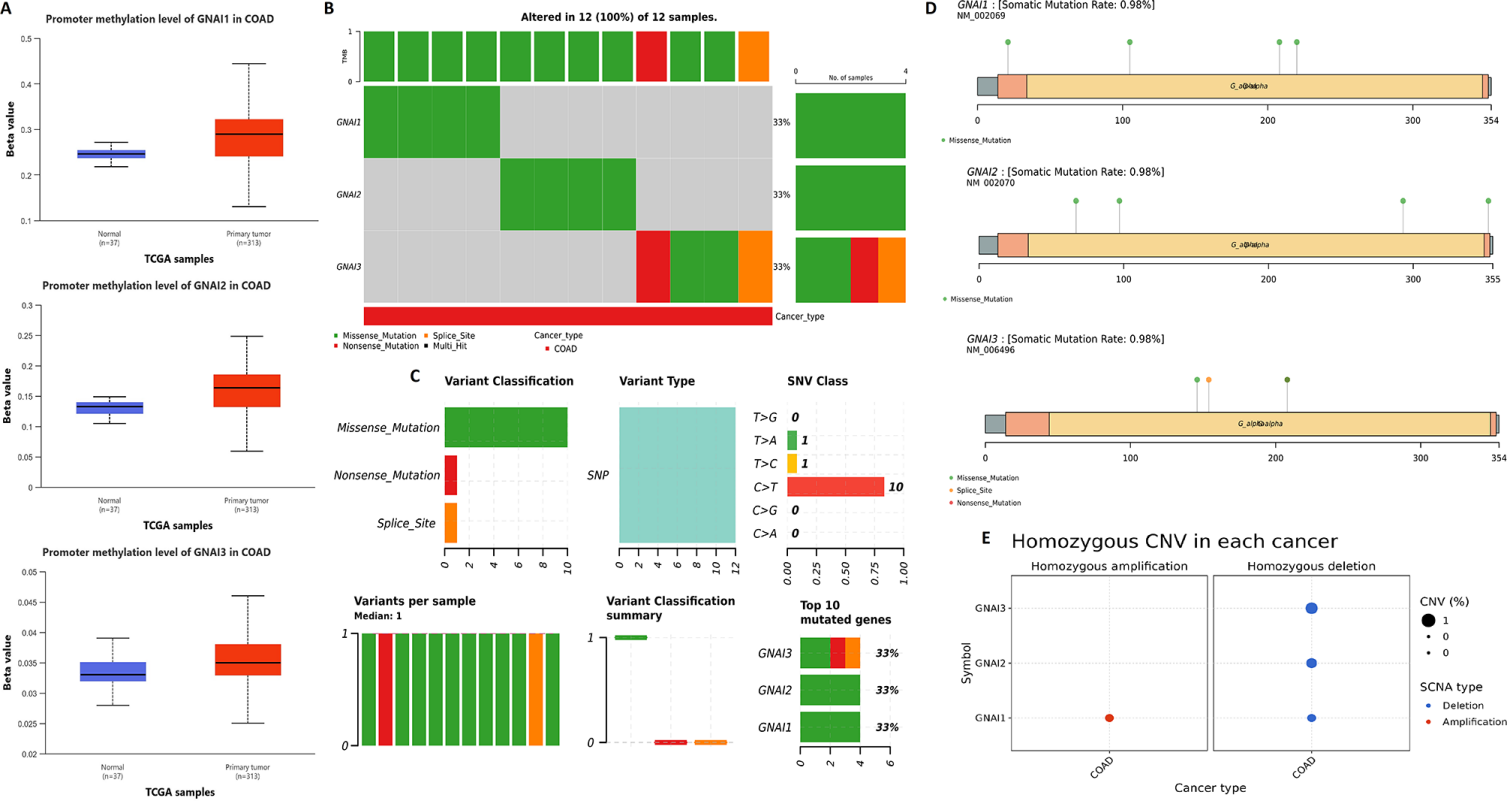
Genetic and epigenetic alterations of GNAI family genes in COAD. (**A**) Promoter methylation levels of GNAI1, GNAI2, and GNAI3 in COAD vs. normal tissues analyzed using UALCAN. (**B**) Oncoplot showing the mutation landscape of GNAI genes in COAD from cBioPortal. (**C**–**D**) Mutation types and frequencies indicate ~ 0.98% somatic mutation rates per gene, with missense mutations being predominant. (**E**) Copy number variation (CNV) analysis from GSCA shows homozygous deletions in GNAI1, GNAI2, and GNAI3, implying genomic loss as a mechanism of downregulation. *P*-value < 0.05

The genetic and epigenetic alterations of GNAI family genes (GNAI1, GNAI2, and GNAI3) in COAD were comprehensively analyzed through promoter methylation, mutation, and copy number variation (CNV) analyses using different databases. Promoter methylation analysis conducted via the UALCAN database revealed significant hypermethylation of GNAI1, GNAI2, and GNAI3 in COAD tumors compared to normal tissues (Fig. 4A). Elevated beta values were observed for all three genes in COAD samples, suggesting that epigenetic silencing through DNA methylation contributes to the downregulation of these genes in COAD (Fig. 4A). Mutation analysis using the cBioPortal database revealed that 33% of COAD samples exhibited mutations in GNAI1, GNAI2, and GNAI3 (Fig. 4B). The majority of these mutations were missense mutations, with somatic mutation rates of 0.98% for each gene (Fig. 4C-D). These findings indicate that mutations in GNAI genes, though low in frequency, are still a significant factor in their altered function in COAD. Additionally, CNV analysis conducted through the GSCA database identified homozygous deletions of GNAI1, GNAI2, and GNAI3 in COAD (Fig. 4E). This suggests that copy number variations, specifically deletions, are involved in the downregulation of these genes, further contributing to their role in cancer progression.

### Survival analysis and pathway regulation of GNAI genes in COAD

**Fig. 5:**
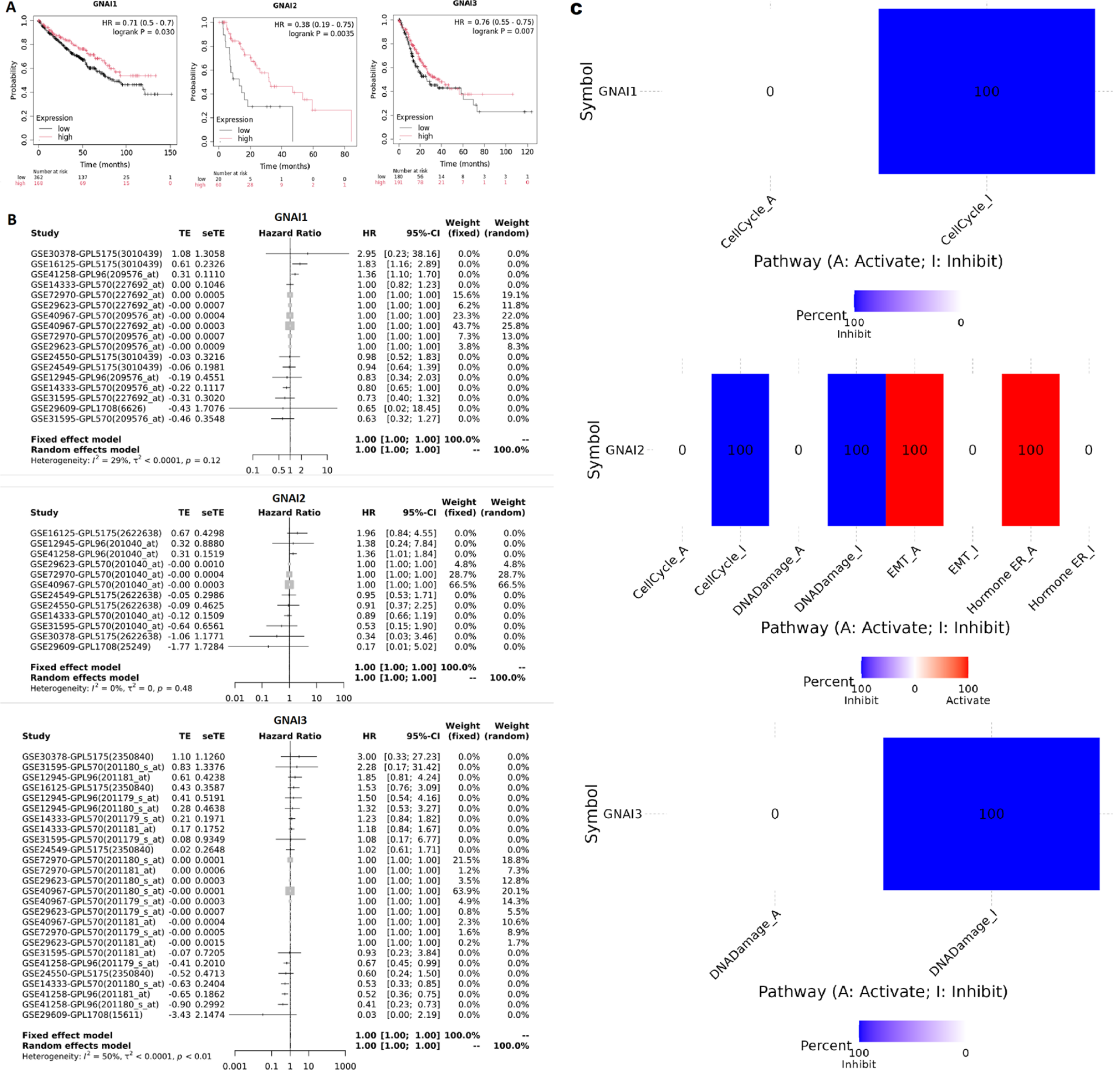
Prognostic significance and pathway regulation of GNAI genes in COAD. (**A**) Kaplan–Meier survival analysis using KM plotter shows that low expression of GNAI1, GNAI2, and GNAI3 was significantly associated with poor overall survival in COAD patients. (**B**) Meta-analysis from the GSCA database reveals hazard ratios (HRs) of 2.16 for GNAI1, 1.42 for GNAI2, and 1.76 for GNAI3, reinforcing their prognostic value across COAD datasets. (**C**) Pathway activity analysis from GSCA shows GNAI1 predominantly inhibits cell cycle and DNA damage repair pathways; GNAI2 is involved in activating EMT and inhibiting estrogen receptor signaling; GNAI3 shows no significant regulatory role in major cancer pathways. *P*-value < 0.05

The survival analysis of GNAI family genes (GNAI1, GNAI2, and GNAI3) was performed using the KM plotter database, which revealed statistically significant associations between their expression levels and overall survival in COAD patients. Specifically, GNAI1 expression showed a significant correlation with survival (log-rank *p* = 0.027), where lower expression was associated with poorer outcomes (Fig. 5A). While GNAI2 and GNAI3 also exhibited trends linking reduced expression to poor survival, the prognostic effects were modest and should be interpreted with caution due to the correlative nature of the analysis. In a supporting meta-analysis using the GENT2 database (Fig. 5B), hazard ratios (HRs) across multiple COAD datasets were calculated: GNAI1 (HR = 2.16) demonstrated the strongest association with disease progression, whereas GNAI2 (HR = 1.42) and GNAI3 (HR = 1.76) showed weaker associations. These HR values indicate a moderate survival impact at best. Taken together, these findings suggest that while GNAI gene expression levels may offer some prognostic relevance, their clinical utility as standalone biomarkers is likely limited. Future work should explore their value as part of a composite risk model and validate their prognostic performance across larger, independent cohorts to establish broader applicability. Finally, the role of GNAI genes in regulating various cancer-associated pathways was explored in Fig. 5C using GSCA database. The results revealed that GNAI1 is primarily involved in the inhibition of cell cycle regulation and DNA damage pathways, with no significant activation detected in these pathways (Fig. 5C). GNAI2 was found to activate EMT pathways and inhibit hormone estrogen receptor (ER) pathways (Fig. 5C). In contrast, GNAI3 showed no significant involvement in the activation or inhibition of these pathways.

### Regulatory role and diagnostic potential of miRNAs targeting GNAI genes in COAD

**Fig. 6:**
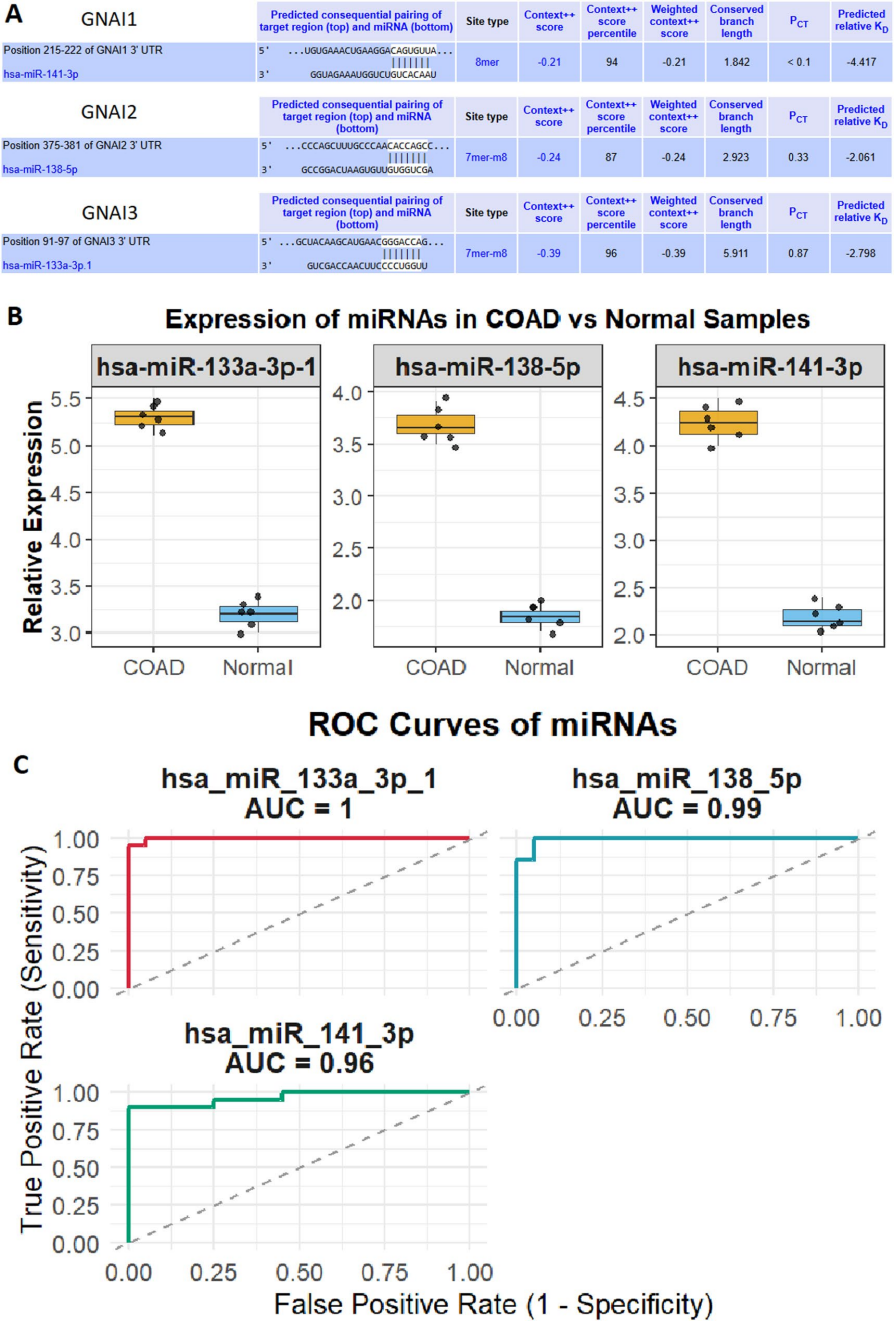
Regulatory role and diagnostic potential of miRNAs targeting GNAI genes in COAD. (**A**) Predicted binding interactions of hsa-miR-133a-3p-1, hsa-miR-138-5p, and hsa-miR-141-3p with the 3′ UTRs of GNAI1, GNAI2, and GNAI3, respectively, identified using the TargetScan database. (**B**) RT-qPCR expression analysis of the miRNAs in nine COAD and six normal cell lines. (**C**) ROC curve analysis showing high diagnostic potential for the miRNAs, with AUCs of 1.00 for hsa-miR-133a-3p-1, 0.99 for hsa-miR-138-5p, and 0.96 for hsa-miR-141-3p. *P*-value < 0.05

miRNA prediction analysis via the TargetScan database revealed that hsa-miR-133a-3p-1, hsa-miR-138-5p, and hsa-miR-141-3p target the 3’ UTR regions of GNAI1, GNAI2, and GNAI3, respectively (Fig. 6A). These miRNAs have strong predicted binding affinities, suggesting their potential regulatory role in GNAI gene expression (Fig. 6A). RT-qPCR analysis of these miRNAs across nine COAD cell lines and six normal control cell lines demonstrated significant upregulation of hsa-miR-133a-3p-1, hsa-miR-138-5p, and hsa-miR-141-3p in COAD cells (Fig. 6B). ROC analysis demonstrated the diagnostic potential of these miRNAs, with hsa-miR-133a-3p-1 achieving an AUC of 1, hsa-miR-138-5p with an AUC of 0.99, and hsa-miR-141-3p showing an AUC of 0.96 (Fig. 6C).

### Immune interactions, infiltration, and drug sensitivity of GNAI genes in COAD

**Fig. 7:**
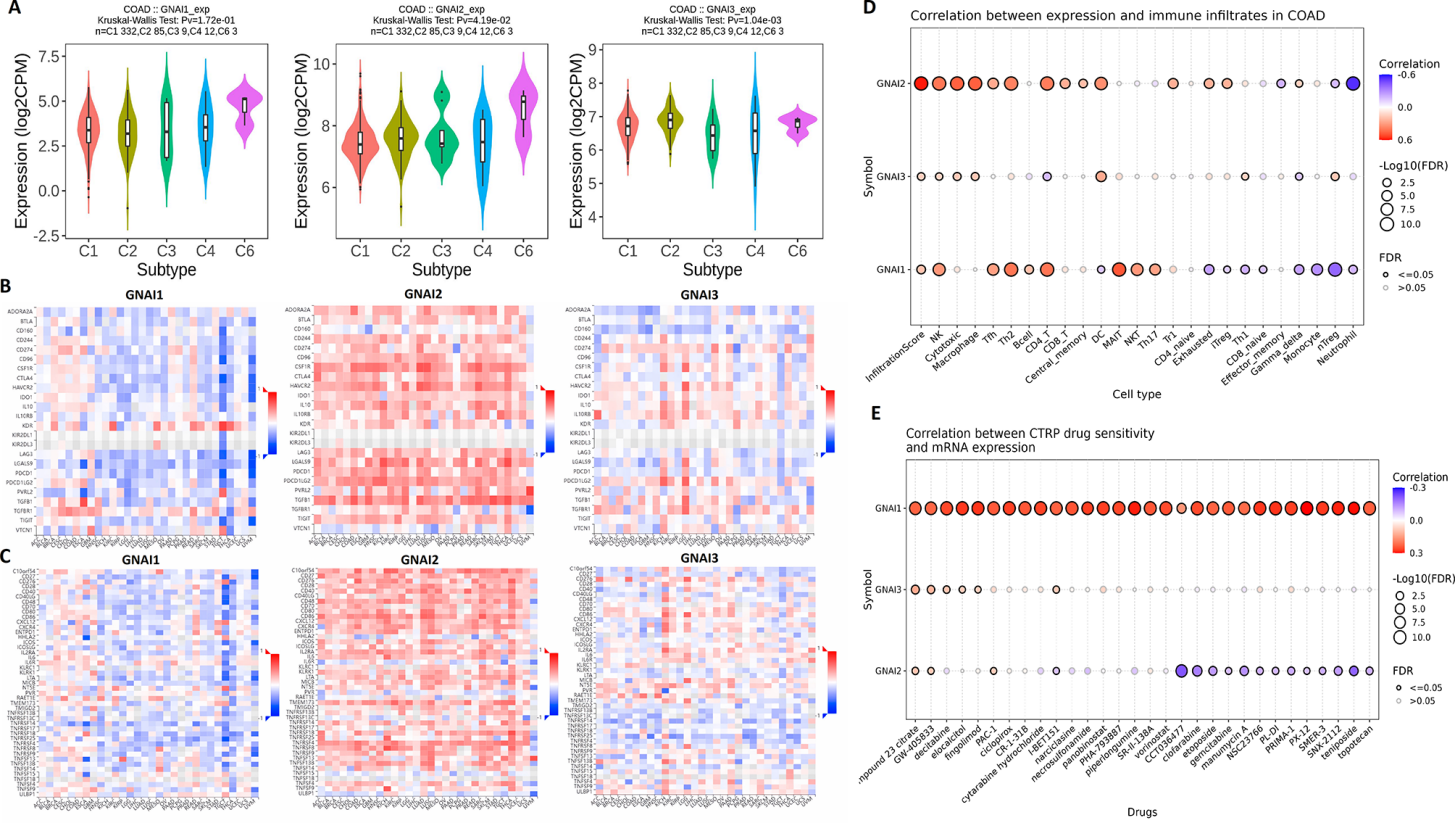
Immune subtype association, immune modulation, and drug sensitivity of GNAI genes in COAD. (**A**) Expression levels of GNAI1, GNAI2, and GNAI3 across COAD immune subtypes from the TISIDB database. (**B**) Correlations between GNAI gene expression and immune inhibitor molecules in COAD. (**C**) Correlations between GNAI gene expression and immune stimulator molecules in COAD. (**D**) Immune infiltration analysis using GSCA shows negative correlations between GNAI gene expression and infiltration of neutrophils, nTregs, and monocytes. (**E**) Drug sensitivity analysis using GSCA indicates that lower expression of GNAI genes was associated with resistance to multiple cancer drugs in COAD. *P*-value < 0.05

The expression of GNAI genes (GNAI1, GNAI2, and GNAI3) was analyzed across different immune subtypes in COAD using the TISIDB database. Significant differences in the expression levels of GNAI genes were observed across various subtypes, with GNAI2 showing higher expression in C2 and C6 immune subtypes, while GNAI1 and GNAI3 displayed variable expression patterns (Fig. 7A). The correlation of GNAI genes with immune inhibitor molecules was analyzed (Fig. 7B). The results indicate that all three GNAI genes are negatively correlated with various immune inhibitors, including PVRL2, KIR2DL3, and TGFB1 etc., suggesting that these genes might play an inhibitory role in immune response regulation in COAD (Fig. 7B). Similarly, the correlation of GNAI genes with immune stimulator molecules was analyzed (Fig. 7C). The findings showed that GNAI genes, particularly GNAI2, was positively correlated with immune stimulators (TNFSF14, TNFSF9, and TNFSF9, etc.), implying a role in promoting immune activation in COAD (Fig. 7C). Immune infiltration analysis conducted using the GSCA database revealed significant negative correlations between the expression of GNAI genes and various immune cell types, including neutrophils, nTreg, and monocytes (Fig. 7D). Lastly, drug sensitivity analysis was performed using the GSCA database. The analysis revealed that lower expression levels of GNAI genes correlate with increased resistance to several cancer drugs (as shown by red color) (Fig. 7E), indicating that GNAI genes may contribute to the chemoresistance in COAD.

### PPI network and functional enrichment analysis of GNAI genes

**Fig. 8:**
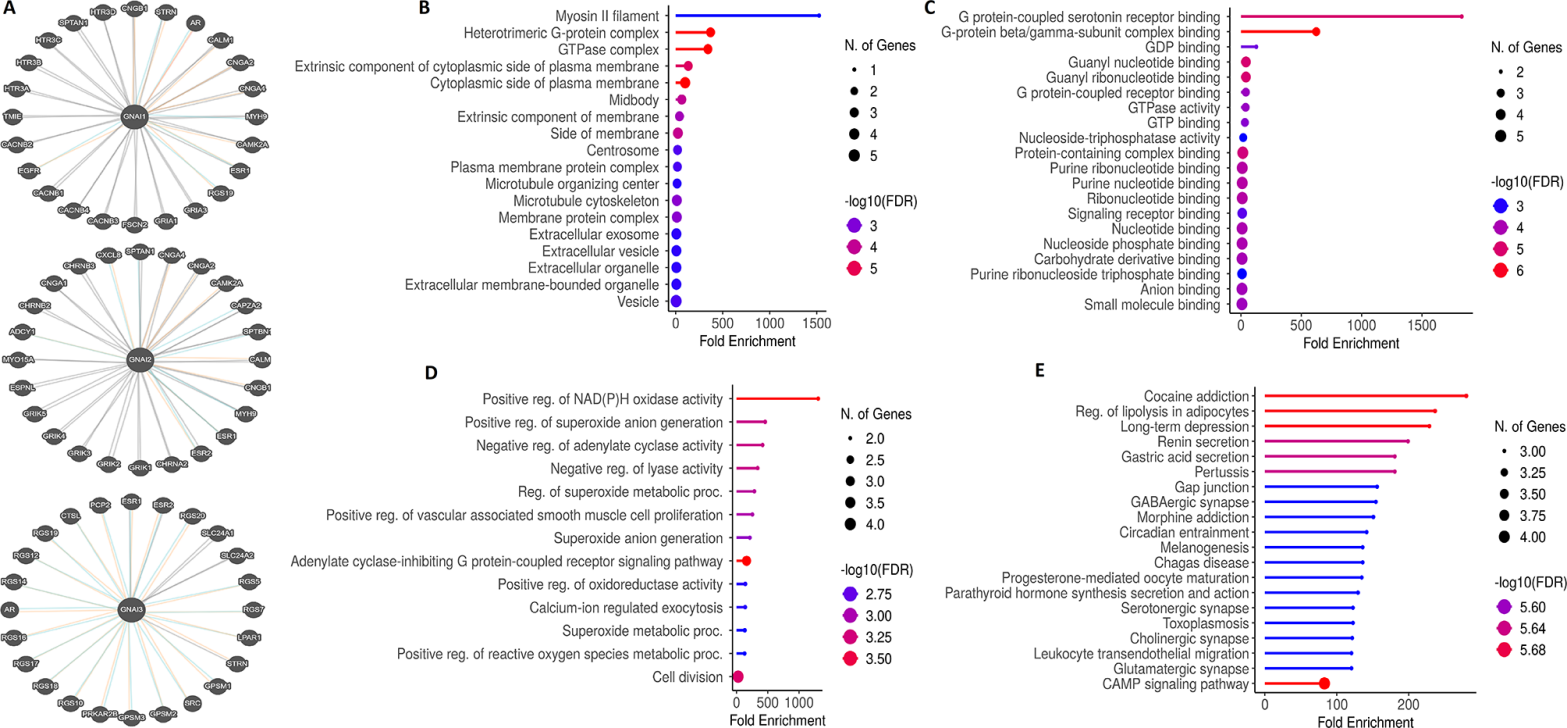
PPI network and functional enrichment analysis of GNAI genes in COAD. (**A**) Protein–protein interaction (PPI) network of GNAI1, GNAI2, and GNAI3 and their associated proteins constructed using the Pathway Commons database, highlighting key interacting partners. (**B**) Cellular component enrichment analysis identifies significant associations with extracellular vesicles, membrane-bounded organelles, and other vesicle-related terms. (**C**) Molecular function analysis reveals enrichment in nucleotide and protein binding activities, including GTP and guanine nucleotide binding. (**D**) Biological process enrichment indicates involvement in redox regulation (e.g., superoxide metabolic processes), NAD(P)H oxidase activity regulation, and cell division. (**E**) KEGG pathway analysis shows enrichment in diverse signaling and metabolic pathways, including cocaine addiction, regulation of lipolysis in adipocytes, and gastric acid secretion, emphasizing the multifunctional roles of GNAI genes. *P*-value < 0.05

The PPI networks of GNAI family genes (GNAI1, GNAI2, and GNAI3) were constructed using the Pathway Commons database. The network visualizations reveal the complex interactions between GNAI genes and various other proteins (Fig. 8A). Gene enrichment analysis was conducted using the DAVID tool to further explore the functional roles of GNAI genes and their binding partners in COAD. Cellular component analysis (Fig. 8B) revealed significant enrichment of terms related to extracellular vesicles, vesicles, and extracellular membrane-bounded organelles, suggesting that GNAI genes are involved in extracellular processes and intercellular communication. Molecular function analysis (Fig. 8C) identified enrichment in several nucleotide-binding functions, including GTP binding, guanine nucleotide binding, and protein complex binding, highlighting the critical role of GNAI genes in cellular signaling and molecular interactions. Biological process analysis (Fig. 8D) revealed significant enrichment in processes such as superoxide metabolic processes, positive regulation of NAD(P)H oxidase activity, and cell division, indicating the involvement of GNAI genes in redox regulation, cell cycle control, and oxidative stress responses. Finally, KEGG pathway analysis (Fig. 8E) showed significant enrichment in pathways related to cocaine addiction, lipolysis regulation in adipocytes, and gastric acid secretion, emphasizing the diverse roles GNAI genes play in both metabolic processes and disease-related pathways.

### Overexpression of GNAI1 and GNAI2 inhibits proliferation, colony formation, and migration in COAD cells

**Fig. 9:**
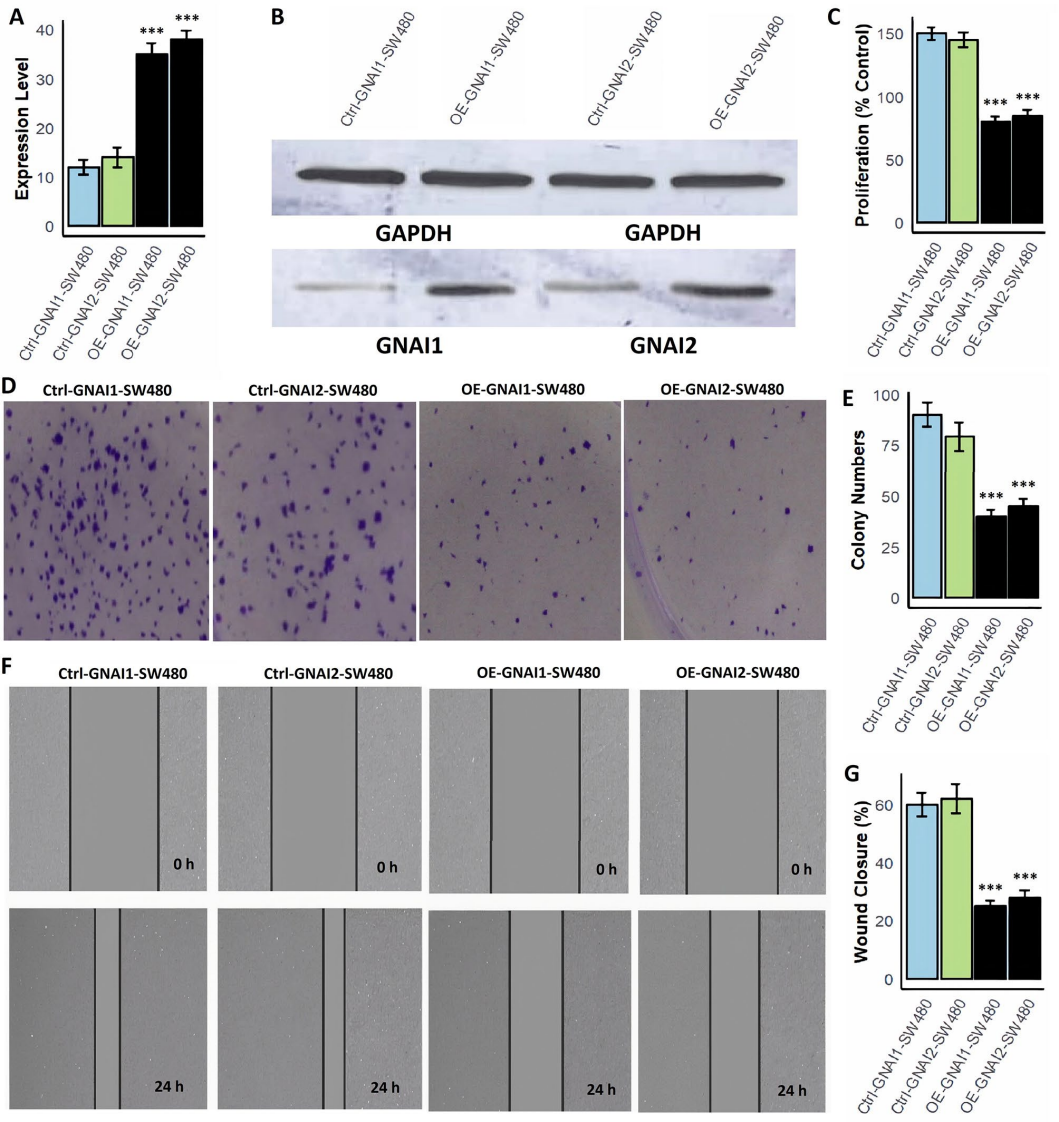
Overexpression of GNAI1 and GNAI2 suppresses proliferation, colony formation, and migration in SW480 COAD cells. (**A**) RT-qPCR validation of GNAI1 and GNAI2 mRNA levels in OE-GNAI1-SW480 and OE-GNAI2-SW480 cells compared to control. (**B**) Western blot analysis confirming increased protein expression of GNAI1 and GNAI2 in overexpression models, normalized to GAPDH. (**C**) Cell proliferation assay shows significantly reduced proliferation in OE-GNAI1 and OE-GNAI2 cells compared to controls. (**D**–**E**) Colony formation assays demonstrating a significant reduction in colony numbers in overexpression groups, indicating impaired clonogenic potential. (**F**–**G**) Wound healing assays show restricted migration capacity in OE-GNAI1 and OE-GNAI2 SW480 cells, with markedly lower wound closure percentages than controls. P***-value < 0.001

**Fig. 10:**
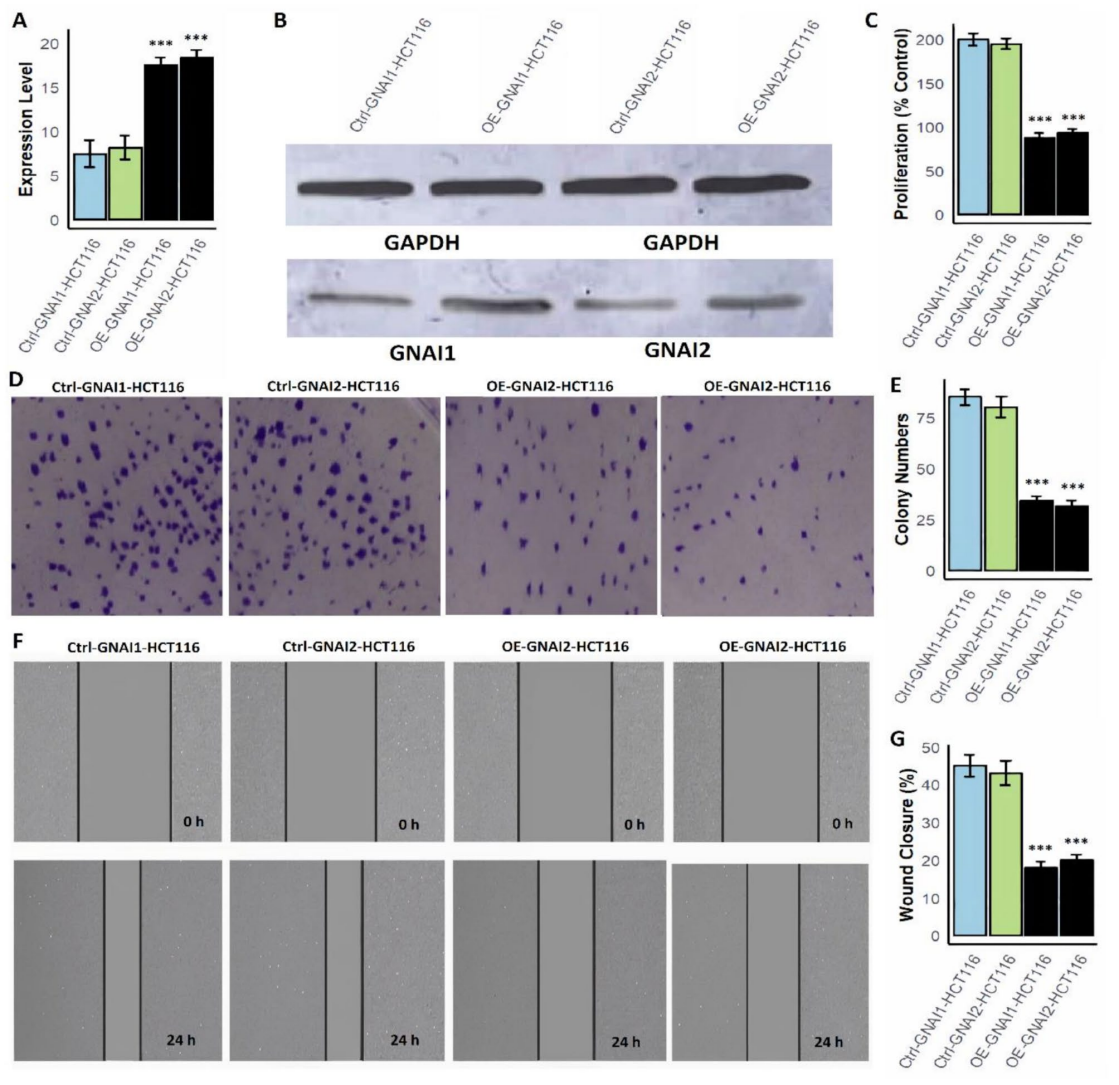
GNAI1 and GNAI2 overexpression inhibits proliferation, colony formation, and migration in HCT116 COAD cells. (**A**) RT-qPCR analysis showing elevated mRNA expression of GNAI1 and GNAI2 in OE-GNAI1-HCT116 and OE-GNAI2-HCT116 cells. (**B**) Western blot confirmation of increased protein expression levels of GNAI1 and GNAI2 in overexpressing HCT116 cells, normalized to GAPDH. (**C**) Cell proliferation analysis revealing significant suppression of proliferation in OE-GNAI1 and OE-GNAI2 groups compared to controls. (**D**–**E**) Colony formation assays show a substantial decrease in colony numbers in both overexpression groups, confirming loss of clonogenicity. (**F**–**G**) Wound healing assays indicate reduced migration capacity in OE-GNAI1 and OE-GNAI2 HCT116 cells with significantly less wound closure over 24 h. P***-value < 0.001

To explore the functional impact of GNAI1 and GNAI2 overexpression in COAD, we employed two COAD cell lines, SW480 and HCT116, and generated overexpression models (OE-GNAI1 and OE-GNAI2) using expression vectors. Functional assays including, mRNA quantification, protein validation, cell proliferation, colony formation, and wound healing, were subsequently performed to evaluate the phenotypic outcomes. RT-qPCR analysis revealed a marked upregulation of GNAI1 and GNAI2 mRNA expression in both OE-GNAI1-SW480/OE-GNAI2-SW480 (Fig. 9A) and OE-GNAI1-HCT116/OE-GNAI2-HCT116 (Fig. 10A) cells compared to their respective control groups. This transcriptional upregulation was further validated at the protein level by Western blotting, which confirmed substantial increases in GNAI1 and GNAI2 protein expression in the overexpression models of both cell lines, normalized to GAPDH (Figs. 9B and 10B and Supplementary data Fig. 1). Functionally, overexpression of either gene led to a significant suppression of cell proliferation. As shown in Figs. 9C and 10C, the OE-GNAI1 and OE-GNAI2 groups exhibited markedly reduced proliferation rates compared to the control groups (*p* < 0.001), suggesting a strong anti-proliferative effect. In the colony formation assays, a consistent pattern was observed. Both OE-GNAI1 and OE-GNAI2 SW480 and HCT116 cells showed significantly fewer colonies than the control groups (Figs. 9D–E and 10D–E), indicating that overexpression of these genes impairs clonogenic survival potential (*p* < 0.001). To further investigate their role in cell motility, wound healing assays were conducted. Control cells displayed a significantly higher percentage of wound closure after 24 h, while OE-GNAI1 and OE-GNAI2 cells showed notably restricted wound closure capabilities (Figs. 9F–G and 10F–G), reinforcing the suppressive role of GNAI1 and GNAI2 in COAD cell migration (*p* < 0.001).

### Overexpression of GNAI1 and GNAI2 modulates EMT marker expression in COAD cells

**Fig. 11:**
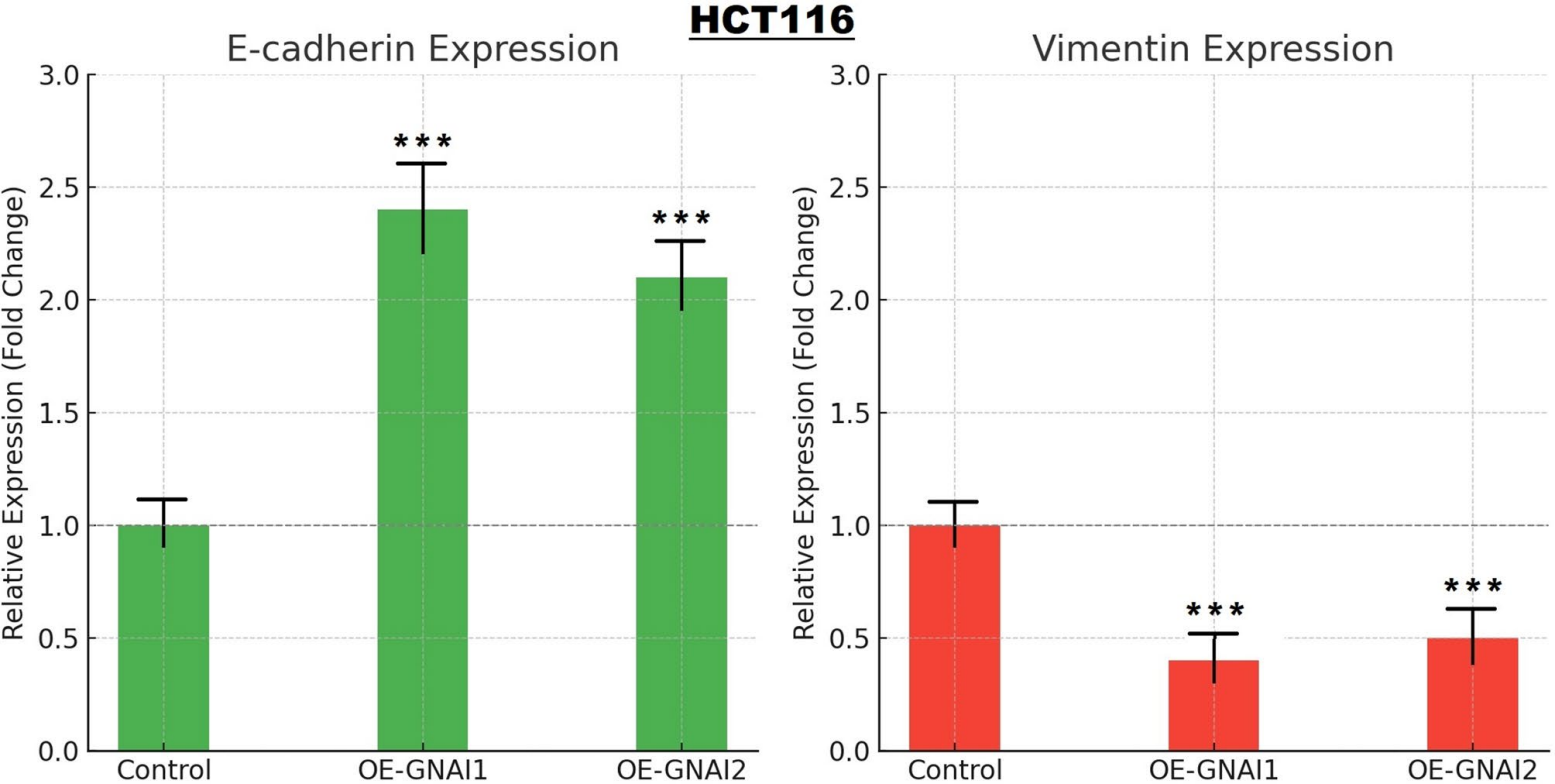
GNAI1 and GNAI2 overexpression alters EMT marker expression in COAD cells. RT-qPCR analysis showing relative mRNA expression levels of E-cadherin (left) and Vimentin (right) in control, OE-GNAI1, and OE-GNAI2 HCT116 COAD cells. P***-value < 0.001

To investigate whether GNAI1 and GNAI2 influence in COAD cells, we examined the expression levels of two canonical EMT markers—E-cadherin (epithelial marker) and Vimentin (mesenchymal marker)—in control, OE-GNAI1, and OE-GNAI2 HCT116 cells using RT-qPCR. As shown in Fig. 11, overexpression of either GNAI1 or GNAI2 led to a substantial upregulation of E-cadherin in transfected cells compared to control cells (Fig. 11). Conversely, Vimentin expression was significantly downregulated in both OE-GNAI1 and OE-GNAI2 cells, indicating suppression of mesenchymal traits (Fig. 11). These findings suggest that GNAI1 and GNAI2 suppress EMT in COAD cells, potentially through the restoration of epithelial characteristics and inhibition of mesenchymal transition. Since EMT is a critical driver of migration, invasion, and metastasis, this observation aligns with our wound healing and colony formation data, where GNAI1/2 overexpression impaired cellular motility and survival.

**Fig. 12:**
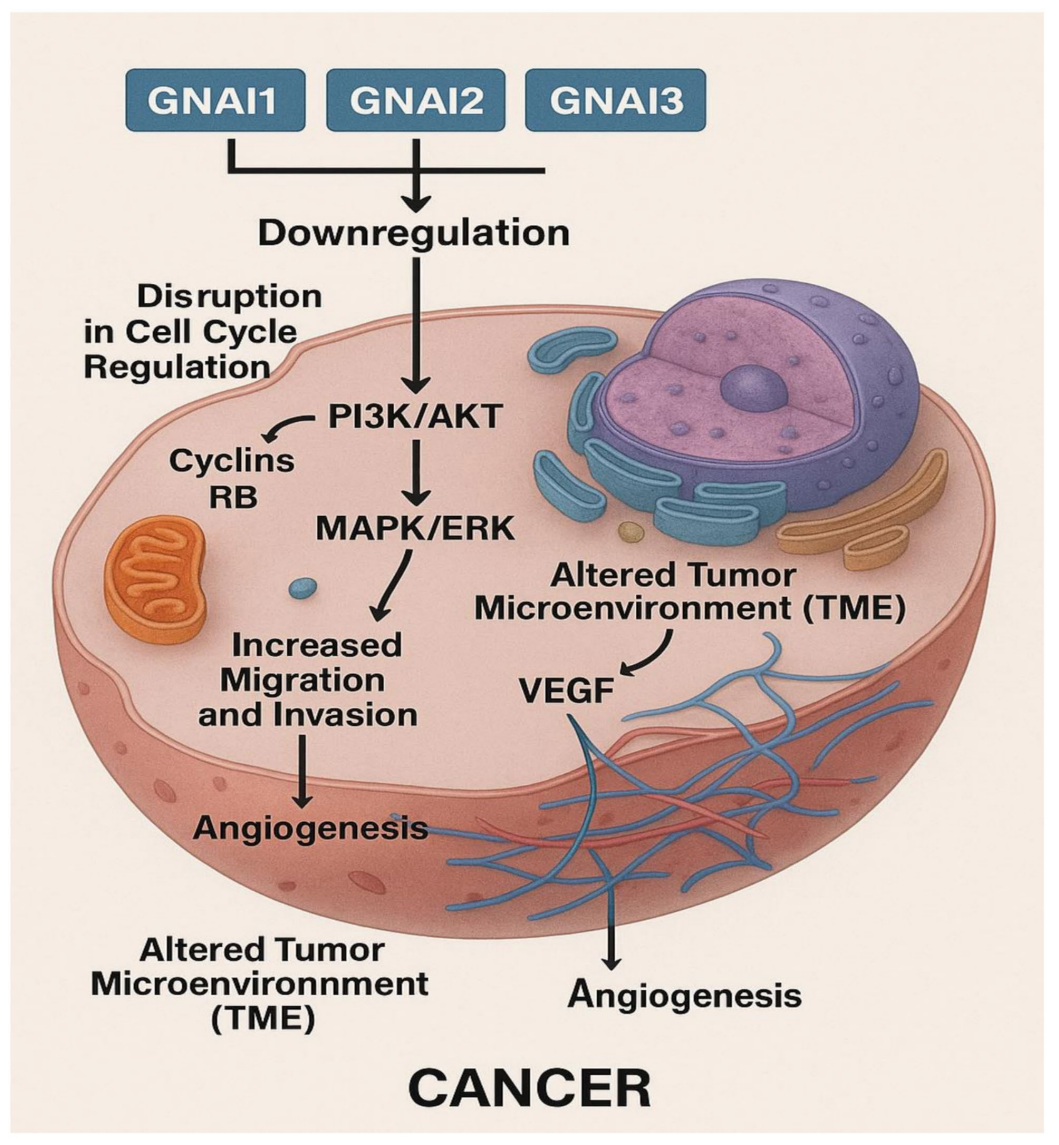
Conceptual pathophysiological model illustrating the consequences of GNAI gene downregulation in COAD. This mechanistic diagram outlines the cellular and molecular alterations resulting from GNAI1, GNAI2, and GNAI3 downregulation. Loss of GNAI signaling leads to disruption of the cell cycle (via Cyclins and RB), promoting uncontrolled G1–S phase progression. Pro-survival pathways such as PI3K/AKT and MAPK/ERK are upregulated, enabling resistance to apoptosis. Simultaneously, enhanced Rho GTPase activity and EMT facilitate cytoskeletal remodeling and increased migration and invasion. Upregulation of VEGF promotes angiogenesis, while immune evasion and tumor microenvironment remodeling support cancer progression. Collectively, these changes highlight the tumor-suppressive roles of GNAI1/2/3 in COAD

Finally, the proposed pathophysiological pathway shows that the loss of GNAI signaling disrupts cell cycle regulation through dysregulation of cyclins and retinoblastoma (RB) protein, resulting in unchecked G1–S phase transition (Fig. 12). Concurrently, pro-survival pathways such as PI3K/AKT and MAPK/ERK are upregulated, promoting cellular proliferation and resistance to apoptosis (Fig. 12). Additionally, the downregulation facilitates EMT and reorganization of the cytoskeleton via Rho GTPase signaling, enhancing cell migration and invasion (Fig. 12). This promotes metastasis. The increased expression of VEGF leads to enhanced angiogenesis, supplying nutrients and oxygen to the growing tumor (Fig. 12). Finally, the tumor microenvironment (TME) is remodeled, favoring immune evasion and supporting tumor progression (Fig. 12). Together, these alterations promote tumorigenesis and malignant transformation, underscoring the tumor-suppressive role of GNAI1/2/3 in COAD. However, further experimental validation—such as western blotting or pathway activation assays targeting key components like p-AKT, p-RB, Cyclin D1, E-cadherin, and N-cadherin—is warranted in future studies to substantiate these proposed mechanisms.

## Discussion

This study was designed based on the hypothesis that members of the GNAI gene family (GNAI1, GNAI2, and GNAI3) play tumor-suppressive roles in COAD and that their downregulation may have diagnostic, prognostic, and therapeutic significance. To test this hypothesis, we conducted integrated in silico and in vitro analyses, combining public datasets with functional validation in COAD cell lines. Our RT-qPCR results demonstrated that GNAI1, GNAI2, and GNAI3 were significantly downregulated in COAD cell lines compared to normal controls. These findings were corroborated by data from the TCGA-COAD cohort accessed via GSCA and OncoDB, and supported by IHC-based protein expression profiles from the HPA database. These results are in line with earlier findings where GNAI1 was shown to suppress hepatocellular carcinoma metastasis via inhibition of the RhoA-ROCK pathway [45], and GNAI2/3 suppressed glioma growth through PI3K/AKT inhibition [46]. Similarly, reduced expression of GNAI3 was linked to immune evasion and poor prognosis in hepatocellular carcinoma [47, 48]. However, none of these studies had previously evaluated GNAI family expression specifically in COAD. Our study is unique in being the first to systematically evaluate all three GNAI genes in COAD using both experimental and computational methods, identifying their robust downregulation and moderate to strong diagnostic potential, especially for GNAI2 (AUC = 0.83).

We further explored mechanisms underlying GNAI gene suppression. Promoter methylation analysis revealed significant hypermethylation of GNAI1, GNAI2, and GNAI3 in COAD samples, while CNV analysis detected homozygous deletions, and mutation analysis indicated a small subset of missense mutations (~ 1%). Hypermethylation of tumor suppressor genes is a well-known mechanism in cancer. Prior research has shown that GNAI2 is hypermethylated in breast cancer [49–53], supporting our findings. However, no previous reports have demonstrated the methylation status or CNV deletions of all three GNAI genes in COAD, making this a novel finding. Thus, our study uniquely demonstrates a combined regulatory mechanism—promoter methylation and copy number loss—underlying the silencing of GNAI genes in COAD.

Kaplan–Meier and meta-survival analyses showed that low expression of GNAI1, GNAI2, and GNAI3 is significantly associated with poor overall survival in COAD. In line with this, previous studies have reported GNAI1 downregulation as a poor prognostic marker in ovarian cancer [54] and GNAI3 suppression associated with metastasis in gastric cancer [55]. However, no prior work has linked GNAI family expression to survival outcomes in COAD. This study is therefore the first to establish prognostic associations for all three GNAI genes in COAD, highlighting their potential utility in survival prediction.

Pathway analysis using GSCA revealed that GNAI1 negatively regulates cell cycle and DNA damage repair pathways, while GNAI2 inhibits hormone ER signaling and activates EMT-related pathways. GNAI3, however, showed no major enrichment in key cancer pathways. Previous studies have shown that GNAI2 regulates EMT in pancreatic and breast cancer via ERK signaling [56, 57], and GNAI1 impacts DNA damage repair via ATM/CHK2 pathway modulation in glioblastoma [58]. While consistent with our findings, the functional roles of GNAI genes in these specific pathways have not been reported in COAD, emphasizing the novelty of this analysis. We identified hsa-miR-133a-3p-1, hsa-miR-138-5p, and hsa-miR-141-3p as potential upstream regulators targeting GNAI1, GNAI2, and GNAI3, respectively. These miRNAs were significantly overexpressed in COAD cell lines and showed excellent diagnostic performance (AUC = 0.96–1.00). miR-133a and miR-138 have previously been shown to suppress tumor suppressor genes in colorectal and lung cancers [59, 60]. However, this is the first report demonstrating these miRNAs’ regulatory roles over GNAI genes in COAD, offering mechanistic insight into their downregulation.

GNAI gene expression showed differential associations across COAD immune subtypes. Notably, GNAI2 was enriched in immune-inflamed (C2 and C6) subtypes and positively correlated with immune stimulators (e.g., TNFSF14), while negatively correlating with immune suppressors (e.g., TGFB1). A few prior studies have implicated GNAI3 in immune cell recruitment and PD-L1 expression regulation in hepatocellular carcinoma [27, 61], but no studies have mapped GNAI genes to immune subtypes or interactions in COAD. This adds a novel immune-oncological dimension to our findings and suggests a role for GNAI2 in modulating anti-tumor immunity.

Our drug sensitivity analysis indicated that low expression of GNAI genes correlates with increased resistance to several anti-cancer agents, suggesting a possible role in chemoresistance. Although GNAI2 has been linked to resistance in trastuzumab-treated breast cancer [62], no previous studies have addressed the association between GNAI gene expression and chemoresistance in COAD, highlighting the novelty of this observation and its therapeutic relevance. Finally, we validated the functional roles of GNAI1 and GNAI2 through overexpression in SW480 and HCT116 COAD cell lines. Overexpression significantly suppressed proliferation, colony formation, and migration, confirming their tumor-suppressive roles. These results are consistent with previous functional studies of GNAI1 and GNAI2 in other cancers [20, 63], but our study is the first to experimentally validate the functional roles of these genes in COAD, supporting their candidacy as therapeutic targets. Although we did not directly investigate the effects of GNAI1 and GNAI2 overexpression on DNA repair pathways, existing sliterature suggest that GNAI1 may modulate DNA damage response signaling, such as the ATM/CHK2 pathway [64], and it is plausible that restoring GNAI1/2 expression in COAD cells may impair DNA repair efficiency, contributing to genomic instability in tumor cells. Similarly, GNAI proteins are known to influence metabolic signaling [65], and their overexpression may potentially suppress glycolysis and alter mitochondrial function, thereby inhibiting the metabolic reprogramming that supports tumor growth. Furthermore, based on our in silico drug sensitivity analyses and prior reports linking GNAI2 to drug resistance in other cancers [55, 66], we speculate that GNAI1 and GNAI2 overexpression could enhance chemosensitivity in COAD cells, possibly by interfering with pro-survival pathways such as PI3K/AKT [67]. Future studies involving metabolic flux analysis, DNA damage assays, and drug treatment experiments are warranted to explore these hypotheses and further elucidate the mechanistic roles of GNAI1 and GNAI2 in colorectal cancer biology and therapy response.

Despite the comprehensive nature of this study, a few limitations should be acknowledged to provide a balanced perspective. First, while our findings were validated across multiple databases and reinforced by in vitro functional assays in two COAD cell lines, the lack of in vivo validation using animal models limits the ability to fully assess the physiological relevance and therapeutic potential of GNAI1 and GNAI2 in a more complex tumor microenvironment. Second, our in vitro experiments, although informative, may not fully capture the heterogeneity of COAD tumors observed in patients. Third, the sample size for certain analyses, particularly those involving miRNA expression and functional validation, was relatively limited, which may affect the generalizability of the results. Fourth, while TargetScan-based predictions were used to identify potential miRNA regulators of GNAI genes, experimental confirmation of these interactions—such as luciferase reporter assays or RNA immunoprecipitation (RIP)—was not performed. As such, the claim of post-transcriptional regulation by miRNAs remains speculative and warrants further investigation. Lastly, although strong associations were observed between GNAI gene downregulation and immune modulation or drug resistance, detailed mechanistic studies are still needed to delineate the specific signaling pathways involved. Future studies incorporating animal models, patient-derived tissues, and direct validation of miRNA–mRNA interactions are essential to confirm these findings and clarify the therapeutic implications of targeting the GNAI family in COAD.

## Conclusion

This study demonstrates that GNAI1, GNAI2, and GNAI3 may act as tumor suppressors in COAD. Their consistent downregulation in COAD tissues and cell lines is linked to promoter hypermethylation, mutations, and copy number deletions. Functional and immune analyses revealed their involvement in immune regulation, cell signaling, and redox processes. Overexpression of GNAI1 and GNAI2 significantly suppressed proliferation, colony formation, and migration in COAD cells, confirming their anti-tumor roles. Additionally, miRNA interactions and drug resistance correlations suggest their potential as diagnostic and therapeutic targets. Together, these findings highlight the clinical significance of GNAI genes in COAD progression and treatment.

## Supplementary Information

Below is the link to the electronic supplementary material.

Supplementary Material 1

## Data Availability

Any type of the data will be provided by the corresponding author.
