# Supplementary figures and images for: A comprehensive in silico and invitro analysis revealed the diagnostic, prognostic and therapeutic potential of GNAI family genes in colon adenocarcinoma (COAD)
Source: Hereditas. 2025 Aug 16;162:162. doi: 10.1186/s41065-025-00523-3 (PMC12357399; doi:10.1186/s41065-025-00523-3)

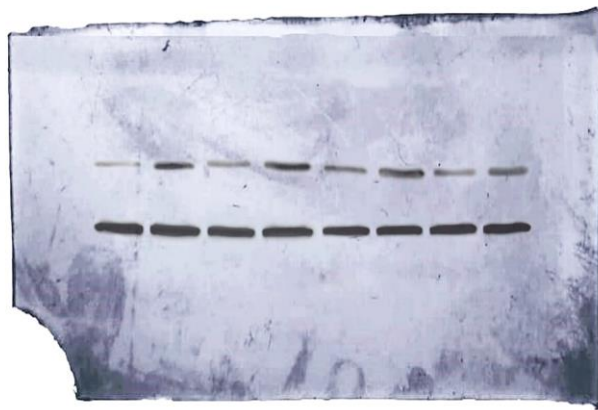

---

**Supplementary data Figure 1: Uncut Western blot bands of GNAI1, GNAI2, and GAPDH.**

Supplement: Supplementary file 1 — Supplementary Material 1 [file 41065_2025_523_MOESM1_ESM.pdf]
